# Coordinated regulation of amino acid metabolism and transport in cereal nitrogen remobilization for grain filling

**DOI:** 10.3389/fpls.2026.1795486

**Published:** 2026-07-08

**Authors:** Shengyan Pang, Xiaotian Ren, Pengjia Wu, Xinhua Lv, Xiang Lin, Dong Wang

**Affiliations:** 1 State Key Laboratory of Crop Stress Resistance and High-Efficiency Production, College of Agronomy, Northwest A&F University, Yangling, Shaanxi, China; 2 Anhui Science and Technology University, Fengyang, Anhui, China

**Keywords:** amino acid interconversion, amino acid permeases, cereal crops, grain filling, nitrogen remobilization, nitrogen use efficiency (NUE), source-sink coordination

## Abstract

Improving nitrogen use efficiency (NUE) in cereals is crucial for sustainable agriculture and global food security. Nitrogen remobilization from senescing leaves to developing grains can contribute up to 80% of grain nitrogen content in cereals, though this varies with genotype and environment, making it a critical determinant of both yield and protein quality. This review synthesizes current evidence to highlight that remobilization efficiency emerges not from isolated processes but from the precise coordination between source leaves and sink grains. Specifically, we discuss how efficient nitrogen remobilization is determined by the coordinated regulation of amino acid interconversion pathways in source leaves—which transform proteolysis products into transport-optimized compounds like glutamine and asparagine—and the spatiotemporally controlled expression of specific amino acid permeases in developing sinks, which govern nitrogen uptake capacity. By synthesizing recent insights into metabolic reprogramming during senescence and transporter expression in grains, we provide a comprehensive overview of how systemic source-sink synchronization optimizes nitrogen flux for grain filling. Building on this synthesis, we hypothesize that engineering the spatiotemporal coordination of amino acid permease expression will yield greater NUE improvements than modulating any single component in isolation.

## 1 Introduction

Ensuring global food security for a burgeoning population hinges on our ability to enhance the productivity and nutritional quality of staple cereal crops. Central to this challenge is the management of nitrogen (N), an essential macronutrient that is often the most limiting factor for grain yield and is the principal determinant of grain protein content. The heavy reliance on synthetic N fertilizers has significant economic and environmental consequences, driving an urgent need to improve the intrinsic nitrogen use efficiency (NUE) of crops (Zhang et al., 2023). A key component of a plant’s NUE is its capacity for nitrogen remobilization—the process of salvaging N from senescing vegetative tissues, primarily leaves, and reallocating it to the developing grains. This internal N economy can contribute over 80% of the total N found in mature grains, making its efficiency a critical trait for both yield and quality.

Historically, research into N remobilization has often adopted a bifurcated approach, focusing either on the catabolic processes within the ‘source’ leaves that supply the nitrogen, or on the transport and assimilation mechanisms within the ‘sink’ grains that determine demand. While these lines of investigation have yielded crucial insights into the component parts—identifying key catabolic enzymes, metabolic pathways, and transporter families—our understanding can be further enriched by examining the dynamic interactions and whole-plant synchronization that ultimately govern the efficiency of the entire process. The central, unresolved question is no longer simply what happens in the source or the sink, but how these two spatially and developmentally distinct domains are precisely synchronized to optimize N flux from senescence to storage.

In this Review, we provide an integrated synthesis of recent molecular evidence that elucidates the specific molecular players and regulatory nodes involved in source-sink coordination. Rather than viewing remobilization through a single rate-limiting step, we highlight that the efficiency of this crucial process is an emergent property of a tightly coupled system. Specifically, we will explore the perspective that the efficiency of nitrogen remobilization for grain filling in cereals is determined by the precisely coordinated regulation of amino acid interconversion pathways in source leaves, which generates a transport-ready N supply, and the spatiotemporally controlled expression of specific transporters, such as amino acid permeases, in developing sinks, which creates the capacity for uptake (Batista-Silva et al., 2019; Wei et al., 2021). We further emphasize that the coordination of amino acid permease spatiotemporal expression in sinks with metabolic conversion rates in sources represents a previously under-appreciated rate-determining interface.

To structure this synthesis, we will first dissect the molecular machinery of ‘The Source Engine: Metabolic Reprogramming for Nitrogen Mobilization’, detailing how senescing leaves are transformed into sophisticated biochemical factories for N salvage and export. We will then shift our focus to the destination, exploring ‘The Sink Machinery: A Coordinated Transport System for Grain Filling’ to understand how the expression of key transporters acts as a critical gatekeeper for N accumulation in the grain. Finally, we will synthesize these two perspectives in ‘Orchestrating Efficiency: Integrating Signals to Tune Source-Sink Dynamics’, examining the complex regulatory networks—involving developmental cues, hormones, and metabolic signals—that synchronize source activity with sink demand and offer new targets for crop improvement.

## 2 The source engine: metabolic reprogramming for nitrogen mobilization

**Figure 1:**
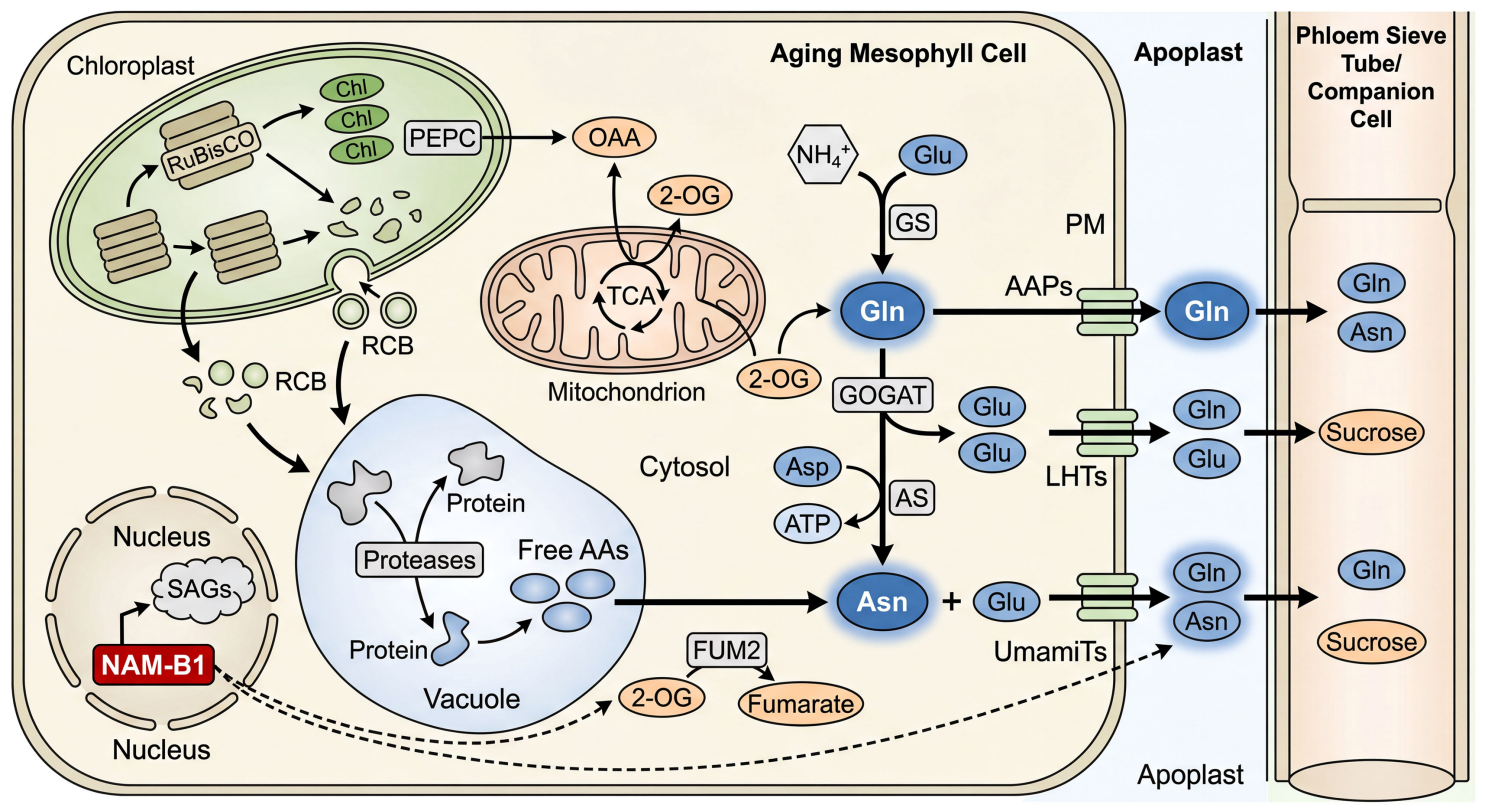
Metabolic reprogramming for nitrogen remobilization in senescing source leaves. The image illustrates the transformation of a leaf cell from a photosynthetic source to a nutrient salvage engine. Protein degradation occurs via autophagy (engulfing chloroplasts) and vacuolar proteolysis, releasing free amino acids. In the cytosol, these amino acids undergo extensive interconversion through the GS/GOGAT cycle to form transport-efficient compounds, primarily Glutamine (Gln) and Asparagine (Asn). This process is fueled by carbon skeletons (2-OG, OAA) derived from mitochondrial TCA cycle activity. The synthesized amino acids are actively loaded into the phloem by transporters such as AAPs and UmamiTs. The entire process is orchestrated by the master transcription factor NAM-B1, which triggers the expression of senescence-associated genes (SAGs). GS, Glutamine Synthetase; GOGAT, Glutamate Synthase; 2-OG, 2-oxoglutarate; OAA, Oxaloacetate; AAPs, Amino Acid Permeases.

The onset of developmental senescence marks a significant functional transition in cereal leaves, converting them from sites of carbon assimilation and amino acid synthesis (**Figure 1**) to highly specialized source tissues for nutrient remobilization. This genetically programmed catabolic process is designed to salvage valuable resources, principally nitrogen (N), for reallocation to developing sinks like the grains (Uauy et al., 2006). The efficiency of this salvage operation—which involves dismantling cellular machinery, converting liberated nitrogenous compounds into transport-efficient forms, and their active export into the vascular network—is a critical determinant of grain protein content and crop quality. This chapter dissects this metabolic reprogramming, a process governed by a hierarchy of regulatory factors, with key transcription factors like NAM-B1 acting as master switches that trigger the downstream cascade of catabolic and transport-related gene expression (Uauy et al., 2006; Gregersen et al., 2008; Barillot et al., 2016; Zhao et al., 2023).

The principal reservoir of nitrogen in a mature leaf is protein, with Ribulose-1,5-bisphosphate carboxylase/oxygenase (RuBisCO) accounting for up to 50% of the total soluble protein in C3 plants. Consequently, the controlled degradation of RuBisCO and other chloroplast proteins is the primary and quantitatively most significant step in N remobilization. This degradation is tightly linked to the decline in photosynthetic activity and chlorophyll content, hallmarks of leaf senescence (Izumi and Hidema, 2015). The process is not a chaotic collapse but an orderly dismantling, primarily mediated by autophagy, where entire chloroplasts or their components are engulfed by the vacuole for breakdown. Concurrently, other cellular proteins stored in the cytoplasm and vacuole are also targeted for proteolysis. This large-scale protein catabolism releases a diverse pool of free amino acids into the cytosol (Liu et al., 2022). Studies on various plant species consistently identify the upregulation of genes associated with protein processing and degradation as a core component of the senescence-associated gene (SAG) expression profile, underscoring the universality of this strategy (Uauy et al., 2006; Distelfeld et al., 2012; Izumi and Hidema, 2015). The resulting increase in the cytosolic amino acid pool, however, is merely the prelude to a more complex phase of metabolic interconversion (**Figure 1**; Gregersen et al., 2008).

The heterogeneous mixture of amino acids liberated from proteolysis is not suitable for direct, efficient long-distance transport. Instead, the senescing leaf cell initiates a major metabolic reprogramming to channel this nitrogen into a few select amino acids, primarily glutamine (Gln) and asparagine (Asn), which possess a high nitrogen-to-carbon (N:C) ratio, making them ideal vehicles for N transport. This metabolic hub is a testament to the plasticity of plant metabolism, shifting from anabolic pathways that build the leaf to catabolic and interconversion pathways that deconstruct it for the benefit of the next generation. The glutamine synthetase/glutamate synthase (GS/GOGAT) pathway, a cornerstone of primary N assimilation, is repurposed during senescence to re-assimilate the large amounts of ammonium released from the catabolism of amino acids like glycine, serine, and histidine. The critical role of GS is highlighted in high-protein rice mutants, which exhibit elevated levels of glutamine synthetase, facilitating a greater flux of nitrogen into transportable forms (Min et al., 2023). The regulation of GS is itself complex, with different isoforms in the roots and leaves responding dynamically to the available nitrogen source and the plant’s overall N status, ensuring that assimilation capacity is matched to supply (Prinsi and Espen, 2015).

The synthesis of these transport amino acids is critically dependent on the availability of carbon skeletons, primarily 2-oxoglutarate (2-OG) and oxaloacetate (OAA), which are supplied by the tricarboxylic acid (TCA) cycle. The tight coordination between C and N metabolism is therefore critical. Mild reductions in the activity of cytosolic NADP-dependent isocitrate dehydrogenase (ICDH), which generates 2-OG, lead to a significant decrease in total amino acid content in tomato, underscoring the direct link between TCA cycle flux and N assimilation capacity (Sulpice et al., 2010). Similarly, the provision of other TCA cycle intermediates, such as fumarate and malate, is crucial. A cytosolic fumarase, FUM2, is required for the massive accumulation of fumarate in Arabidopsis leaves, a process that is essential for rapid growth under high nitrogen conditions, likely by providing a carbon sink and source to balance N assimilation (Pracharoenwattana et al., 2010). The importance of this C-N interplay is further demonstrated in wheat, where high nitrogen use efficiency (NUE) near-isogenic lines exhibit enhanced TCA cycle activity and a greater capacity for amino acid synthesis in response to nitrogen application, linking an efficient source metabolism to improved overall N economy (Zhang et al., 2023).

Beyond the biochemical coordination of these pathways, their spatial organization is also a key feature of their efficiency. Proteomic analysis of maize, a C4 plant, reveals a sophisticated division of labor, with enzymes for the synthesis of certain amino acids, such as branched-chain amino acids, being enriched in mesophyll chloroplasts, while others are concentrated in bundle sheath chloroplasts, illustrating a highly structured metabolic network designed to optimize carbon and nitrogen flow (Friso et al., 2010). In some species, such as rice, this compartmentation is even more intricate, with a unique, intrinsically located chloroplastic phosphoenolpyruvate carboxylase (PEPC) playing a crucial role in providing OAA directly within the chloroplast for ammonium assimilation, a specialization that is particularly advantageous under ammonium-rich conditions (Masumoto et al., 2010).

The final step within the source engine is the efficient export of the synthesized transport amino acids from the mesophyll cells into the phloem for long-distance allocation. This process is not passive diffusion but is mediated by a suite of plasma membrane-localized transporters. The Amino Acid Permease (AAP) family of transporters are key players in this phloem-loading step. Transcriptomic studies in pea (Pisum sativum) have shown that the expression of specific AAP genes is significantly upregulated in mature and senescing leaves, coinciding with the period of maximal N remobilization (Ninan et al., 2019). The functional importance of these transporters is unequivocal. Overexpression of a pea AAP, PsAAP1, in both source leaves and developing embryos, resulted in a significant increase in N transport to the seeds, leading to enhanced seed protein content and significant increases in biomass (33%) and seed yield (Zhang et al., 2015). This provides compelling evidence that the transport capacity for amino acids at the phloem-loading stage can be a major rate-limiting step for N remobilization. Similarly, in rice, the altered expression of OsAAP3 was shown to significantly impact arginine transport, leading to premature leaf senescence, demonstrating that the misregulation of a single transporter can disrupt the entire source-sink balance (Wei et al., 2021).

Beyond the AAPs, other transporter families, such as the Lysine-Histidine-Transporter (LHT) family and the recently characterized UmamiT family of amino acid exporters, also contribute to this efflux, creating a robust and potentially redundant system for phloem loading (Gratz et al., 2021; Cao et al., 2025). The expression of these transporters is, in turn, responsive to the plant’s nutritional status. For instance, several AAP genes in tea plants are induced under nitrogen deficiency, suggesting a mechanism to enhance N scavenging and reallocation when external supply is limited (Li et al., 2020).

The precise regulation of this entire metabolic engine, from protein breakdown to phloem loading, is governed by a complex integration of developmental and metabolic signals. The cellular carbon-to-nitrogen (C/N) ratio acts as a critical metabolic sensor. A high C/N ratio, often resulting from declining photosynthetic capacity during senescence, is a potent signal to initiate N remobilization. The intimate link between carbon and nitrogen status is highlighted by studies showing that manipulation of sucrose transport directly impacts nitrogen metabolism and partitioning. For example, overexpressing sucrose transporters to enhance C export from leaves can concurrently boost N assimilation and amino acid transport, suggesting that sink demand for carbon can pull nitrogen through the system (Lu et al., 2020). In rice, the transcription factor OsDOF11 has been shown to affect nitrogen metabolism by modulating the expression of sucrose transporters, providing a direct molecular link between C transport signaling and N-use phenotypes (Huang et al., 2021).

Synthesizing the current literature reveals a functional hierarchy among these components regarding their relative importance as rate-determining steps for nitrogen use efficiency (NUE). At the apex of this regulatory cascade are transcription factors; among them, NAM-B1 possesses the most robust experimental evidence in cereals, acting as a master switch whose manipulation exerts profound, system-wide effects on nitrogen mobilization. Downstream of this transcriptional control, the metabolic capacity of the GS/GOGAT cycle represents a primary and well-substantiated biochemical bottleneck. The flux through this cycle directly dictates the sheer volume of nitrogen that can be successfully converted into transport-efficient forms. Finally, at the export interface, phloem-loading capacity mediated by AAPs represents a critical, yet less comprehensively characterized, rate-limiting step. While overexpression studies underscore the potential of AAPs to enhance sink delivery, their relative contribution to whole-plant NUE under complex field conditions requires further *in vivo* validation. Importantly, this hierarchy of rate-limiting steps is not absolute; current evidence suggests it is heavily genotype- and environment-dependent, which constitutes a major area for future investigation.

In summary, the senescing leaf operates as a regulated biochemical system sequentially executing protein degradation, metabolic funneling, and active phloem loading. Yet, the generation of this supply represents only one half of the source-sink equation. The ultimate success of nitrogen remobilization hinges on the capacity of the sink tissues to effectively receive, unload, and utilize this vital nutrient stream, a topic that will be explored in the subsequent section.

## 3 The sink machinery: a coordinated transport system for grain filling

The efficient generation of a nitrogen-rich phloem stream by the source leaf is only the first act of nutrient remobilization. The ultimate determinant of grain protein content and yield is the sink’s capacity to attract, import, and assimilate this nutrient supply. The grain is not a passive receptacle but an active, highly competitive metabolic entity whose ability to draw in resources defines its Sink Strength. This strength is fundamentally a function of transport activity, governed by a multi-step, sequential transport system that moves amino acids from the maternal vasculature into the filial endosperm and embryo. This journey involves a series of regulated gates, from phloem unloading in maternal tissues to active uptake by the endosperm, creating a transport continuum where the expression and activity of specific membrane transporters act as critical rate-limiting checkpoints. This section deconstructs the machinery of the sink, revealing how the coordinated expression of transporters and metabolic enzymes within the grain dictates the final efficiency of nitrogen utilization for grain filling.

The journey of amino acids into the grain begins with their exit from the plant’s long-distance transport highway, the phloem. This process, termed Phloem Unloading, occurs within the maternal tissues that envelop the developing seed, such as the nucellar projection in cereals. In many developing seeds, there is a lack of symplastic continuity—a break in the network of plasmodesmata—between the maternal tissues and the filial tissues (the endosperm and embryo). This symplastic isolation necessitates an apoplastic step, where assimilates are released from the maternal cells into the extracellular space before being taken up by the filial cells. This apoplastic transfer is a crucial control point, transforming a continuous symplastic flow into a regulated, two-step process of export and import. The initial export of amino acids from the maternal phloem and surrounding parenchyma cells into the apoplast is an active, carrier-mediated process. Members of the UmamiT family of amino acid exporters are prime candidates for mediating this efflux, releasing the nitrogenous cargo into the maternal-filial apoplastic domain (Karmann et al., 2018). This regulated release establishes the concentration and composition of the amino acid pool in the apoplast, which serves as the direct nutrient source for the developing endosperm and embryo.

Once released into the apoplast, amino acids diffuse across the physical gap separating the maternal and filial generations. This apoplastic space, though seemingly a simple conduit, represents a critical interface where the sink exerts its “pull.” The concentration of amino acids in this compartment is a dynamic equilibrium, balanced by the rate of unloading from the maternal side and the rate of uptake by the filial side. The efficiency of this transfer is therefore contingent on the sink’s ability to create and maintain a steep concentration gradient by rapidly importing the available amino acids. This active uptake by the filial tissues is the primary engine of sink strength and the most significant bottleneck in the nitrogen transport continuum.

The active import of amino acids from the apoplast into the filial tissues, particularly the specialized transfer cells of the endosperm and the developing embryo, is the central mechanism defining sink capacity. This process is mediated by a suite of high-affinity importers located on the plasma membranes of these cells. The Amino Acid Permeases (AAPs) are a major family of transporters that play a central role in this uptake. Their expression is often spatially and temporally fine-tuned to coincide with the peak period of Grain Filling. For instance, studies comparing peanut cultivars with differing seed protein contents reveal that the expression profiles of genes involved in amino acid metabolism and transport are key determinants of the final amino acid accumulation (Li et al., 2022). High-protein cultivars often exhibit distinct expression patterns of transporter genes, underscoring that the genetic architecture controlling sink uptake capacity is a primary driver of seed nutritional quality. This principle is further supported by integrated analyses in peanut, which have identified specific transporter genes and transcription factors within gene co-expression modules that are highly correlated with protein content, highlighting a coordinated regulatory network that governs sink activity (Li et al., 2024). The localization of these transporters is also critical; they are frequently concentrated in transfer cells, which possess elaborate cell wall invaginations that significantly increase the surface area of the plasma membrane, thereby maximizing the capacity for solute import from the apoplast (O'Neill and Lee, 2020; Tiozon et al., 2026).

**Figure 2:**
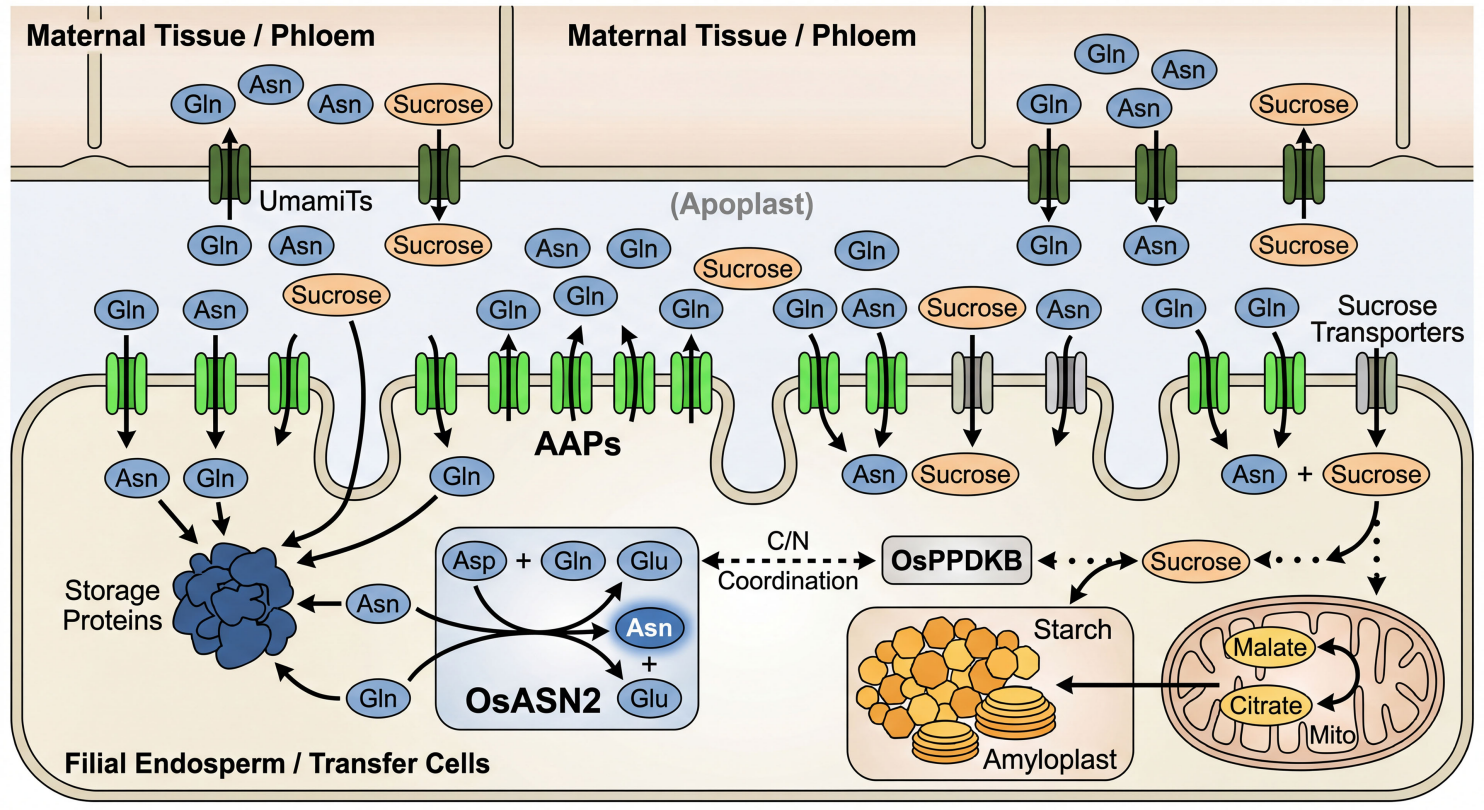
Coordinated transport and assimilation mechanisms in developing grains. The diagram depicts the molecular pathway of nitrogen transfer from maternal tissues to the filial endosperm across the apoplastic barrier. Amino acids (Gln, Asn) are unloaded from the maternal phloem/vascular bundle via efflux transporters (e.g., UmamiTs) into the apoplastic space. Specialized transfer cells in the filial endosperm enhance uptake capacity through extensive cell wall invaginations and high-affinity AAPs. Inside the endosperm, nitrogen assimilation is tightly coupled with carbon metabolism. The enzyme OsASN2 converts imported amino acids into storage protein precursors, while OsPPDKB coordinates the carbon flow for starch synthesis. This metabolic sink strength maintains the concentration gradient required for continuous nutrient import. AAPs, Amino Acid Permeases; OsASN2, Asparagine Synthetase 2; OsPPDKB, Pyruvate Phosphate Dikinase B.

The function of the sink, however, extends beyond mere import. Once inside the endosperm cells, the imported carbon (as sucrose) and nitrogen (as amino acids) are channeled into a complex metabolic network for assimilation and storage (**Figure 2**). The transported amino acids, primarily glutamine and asparagine, are rapidly metabolized. This metabolic conversion serves two purposes: it provides the diverse array of amino acids required for the synthesis of storage proteins, and it maintains a low intracellular concentration of the transport amino acids, thereby sustaining the concentration gradient that drives continued import. The critical nature of this internal metabolic machinery is starkly illustrated in rice. The knockout of a single asparagine synthetase gene, OsASN2, which is crucial for processing imported asparagine within the sink, results in severely defective endosperm development, aberrant starch accumulation, and a complete failure of seed germination (Hu et al., 2025). This demonstrates unequivocally that the capacity to metabolize imported nitrogen is as important as the capacity to import it. The metabolic landscape within the endosperm is complex, with intricate cross-talk between different amino acid pathways. For example, engineering high-lysine rice has been shown to unexpectedly trigger the accumulation of serotonin, revealing a metabolic link between lysine catabolism, stress signaling pathways, and secondary metabolism within the endosperm (Yang et al., 2018). This highlights that the sink is not a simple storage depot but a dynamic biochemical reactor where the flux of imported nutrients is channeled through a complex and interconnected metabolic network.

As in the source leaves, the metabolic activities of the sink are governed by the tight coupling between carbon (C) and nitrogen (N) metabolism. The synthesis of storage proteins is an energy-intensive process that demands a constant supply of both amino acid building blocks (N) and carbon skeletons, which are derived from the catabolism of sucrose imported via the phloem. The coordination between these two streams of resources is critical for efficient grain filling. The interaction between the rice asparagine synthetase OsASN2 and the pyruvate phosphate dikinase OsPPDKB, a key enzyme in C4 photosynthesis that also functions in sucrose-to-starch conversion in rice endosperm, provides a direct molecular link between N and C metabolism within the sink (Hu et al., 2025). Perturbations in carbon metabolism can have direct consequences on amino acid pools and sink function. In Arabidopsis, the disruption of a plastidial fructose-bisphosphate aldolase (FBA) isoform primarily expressed in heterotrophic tissues like roots leads to significant reductions in the levels of branched-chain and aromatic amino acids, demonstrating that defects in central carbon metabolism within a sink organ can directly impair its ability to synthesize essential compounds (Carrera et al., 2021). Furthermore, the metabolic flexibility of the sink relies on the sophisticated compartmentation of pathways. The exchange of organic acids like malate and citrate across the mitochondrial membrane, mediated by specific carriers, is crucial for balancing the TCA cycle, providing carbon skeletons, and maintaining redox homeostasis, all of which are essential for supporting high metabolic rates in sink tissues (Lee et al., 2021).

The entire sink machinery, from transporter expression to metabolic enzyme activity, is under tight genetic and hormonal control, and exhibits significant natural variation. The expression of key transporter genes is regulated by a combination of developmental programs and metabolic signals, including the availability of sugars and amino acids themselves, allowing the sink to adjust its uptake capacity in response to the supply from the source. The genetic basis for this variation is a key target for crop improvement. While foundational insights into nitrogen partitioning and long-distance signaling have often been pioneered in dicot models—such as the correlation of amino acid metabolic gene expression with N distribution in tobacco (Li et al., 2025) or the use of grafting to uncover systemic hormonal and metabolic regulation (Niu et al., 2025)—these principles provide critical blueprints for understanding cereal systems. Similarly, the plant’s response to other nutrient limitations, such as phosphate, involves a significant reprogramming of N metabolism, indicating a complex cross-talk between nutrient signaling pathways that ultimately influences the balance between nitrogen supply and demand (Alexova et al., 2017). Translating these dicot-derived concepts to monocots is essential. For instance, the long-distance signaling mechanisms revealed by grafting in tobacco strongly suggest that manipulating analogous phloem-mobile signals in wheat or rice could significantly alter nutrient utilization efficiency, representing an untapped potential for cereal crop improvement (Niu et al., 2025).

In conclusion, the developing grain functions as a highly sophisticated and active sink, whose capacity to import and process nitrogen is a decisive factor in determining crop yield and quality. The journey of amino acids from the phloem into the endosperm is a tightly regulated, multi-step process mediated by a succession of specific transporters acting as gatekeepers. The expression of these transporters, particularly AAPs at the site of filial uptake, represents a primary bottleneck and a key determinant of sink strength. However, import capacity alone is insufficient; it must be matched by a robust metabolic capacity within the sink to convert transport amino acids into storage products, a process that relies on the intimate coordination of carbon and nitrogen metabolism. Significant knowledge gaps remain regarding the precise molecular signals that communicate sink demand and coordinate the expression of the vast array of transporters and enzymes involved. The complex, genotype-specific spatial organization of metabolites within tissues suggests that simply manipulating a single gene may be insufficient to enhance overall efficiency (Liu et al., 2016; Yang et al., 2018). The metabolic reprogramming required is often extensive and involves entire networks of genes, as seen in the response of tea plants to environmental stress (Zhang et al., 2017). Understanding this integrated system—how the source engine and the sink machinery are synchronized at the whole-plant level—is the final piece of the puzzle. The next section will explore the signaling networks that govern these complex interactions, tuning resource allocation to optimize nitrogen remobilization.

## 4 Orchestrating efficiency: integrating signals to tune source-sink dynamics

**Figure 3:**
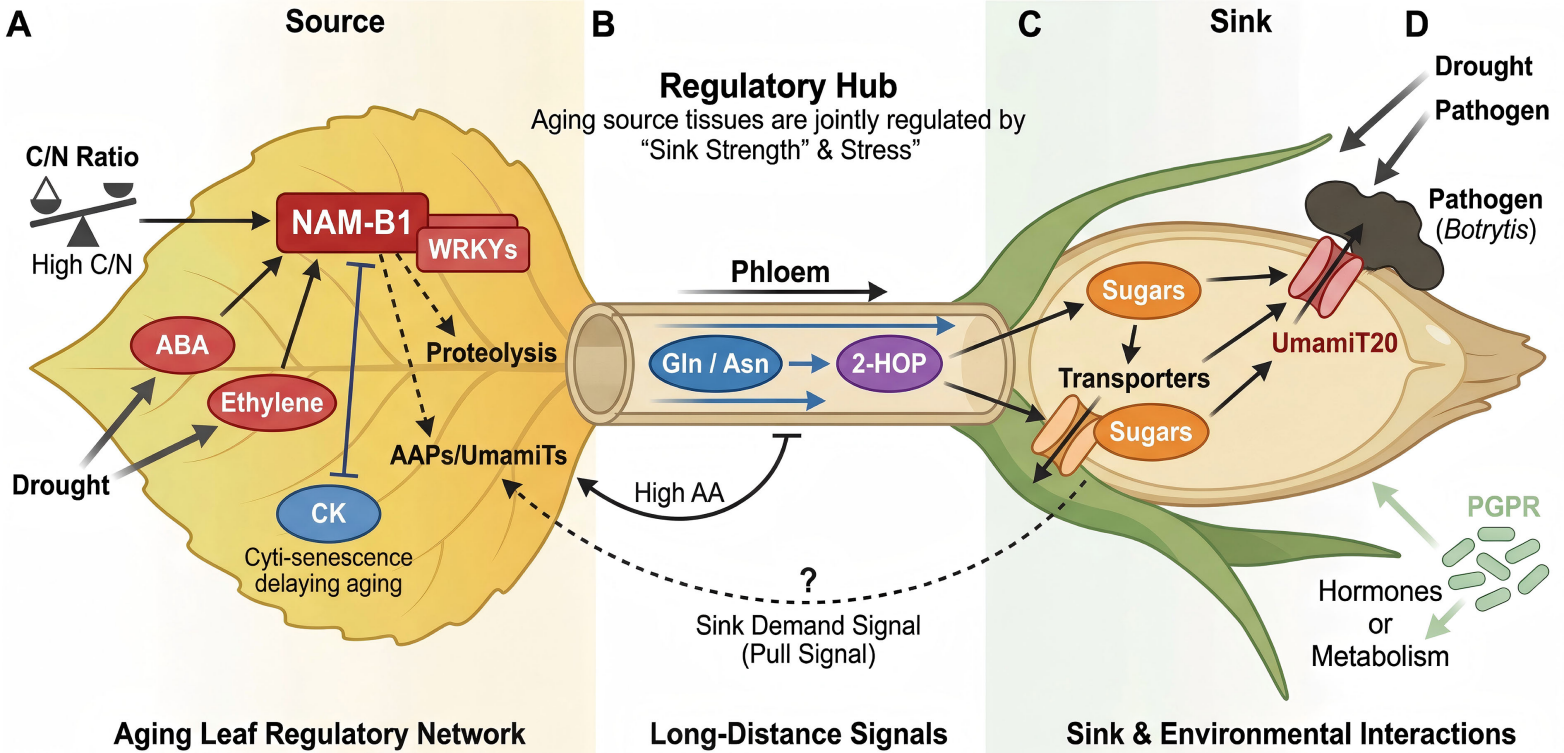
An integrated signaling network orchestrating source-sink nitrogen dynamics. A whole-plant model demonstrating the systemic regulation of Nitrogen Use Efficiency (NUE). **(A)** The Source Hub: In senescing leaves, the master regulator NAM-B1 integrates developmental cues and metabolic signals (e.g., C/N ratio). Senescence is accelerated by ABA and Ethylene (ET) but delayed by Cytokinins (CK), creating a finely tuned “growth vs. survival” trade-off. **(B)** Phloem Signaling: Long-distance signals, including sugars and specific metabolites (e.g., 2-HOP), communicate sink demand back to the source. High amino acid concentrations in the phloem can trigger negative feedback to inhibit further export. **(C)** Sink & Environment: Environmental stresses (Drought, Pathogens) and beneficial interactions (PGPR) act as external inputs that modulate the network, influencing transporter expression (e.g., UmamiT20 under pathogen attack) and shifting the balance between remobilization and storage. ABA, Abscisic Acid; CK, Cytokinin; 2-HOP, 2-hydroxy-5-oxoproline; PGPR, Plant Growth-Promoting Rhizobacteria.

The preceding sections have deconstructed the nitrogen remobilization process into two principal components: a source engine that generates a transport-ready supply of amino acids and a sink machinery that actively imports and utilizes this supply. However, the remarkable efficiency of this system in cereals does not arise from the independent operation of these modules. Rather, it is the product of a dynamic regulatory network that precisely coordinates their activities in time and space. This network functions as a central control panel, integrating a multitude of internal and external signals to modulate the rate and duration of nitrogen flow from source to sink. It ensures that the catabolic activity of the source is synchronized with the anabolic demands of the sink, a balance that is critical for maximizing grain protein content and overall yield. This final chapter synthesizes our understanding of this integrated system, exploring how developmental programs, hormonal cascades, metabolic feedback, and environmental perturbations converge to synergistically drive the efficiency of nitrogen remobilization (**Figure 3**).

Developmental cues initiate and entrain the remobilization program, with leaf senescence acting as the primary trigger. This is not a passive decline but a genetically encoded process initiated by master regulatory transcription factors, such as the well-characterized `NAM-B1` in wheat. These transcription factors act as high-level switches that initiate a downstream cascade of gene expression, activating the catabolic enzymes responsible for protein degradation in the source leaf and, critically, the transporters required for both phloem loading in the source and unloading in the sink. The expression of these master regulators is itself tightly linked to the plant’s life cycle, ensuring that the dismantling of the leaf factory is timed to coincide with the peak demand from the developing grain. The conservation of this developmental program across different plant species underscores its fundamental importance (Uauy et al., 2006; Distelfeld et al., 2012; Andleeb et al., 2023). However, the precise timing and rate of this program are not fixed; they are continuously modulated by a complex interplay of hormonal signals that act as fine-tuning dials on the central control panel.

To understand the complexity of this regulatory hub, the current evidence regarding systemic signaling can be categorized into three distinct levels based on mechanistic understanding: well-established regulatory processes, experimentally supported but mechanistically incomplete feedback loops, and speculative long-distance signals.

Firstly, among the well-established regulatory processes, hormonal cascades and global carbon-to-nitrogen (C/N) ratio sensing form the foundational layer of source-sink coordination. Senescence-promoting hormones, such as abscisic acid (ABA) and ethylene, play a pivotal role in accelerating the remobilization process. ABA, in particular, is a key integrator of stress signals, and its accumulation during senescence or drought stress has been shown to upregulate genes involved in protein catabolism and amino acid transport (Rattanakan et al., 2016). Proteomic and phosphoproteomic analyses have revealed that ABA can rapidly induce widespread changes in protein abundance and phosphorylation status, affecting processes from photosynthesis to membrane transport, thereby remodeling cellular metabolism to favor nutrient salvage (Rattanakan et al., 2016). Conversely, senescence-delaying hormones like cytokinins (CKs) act as a brake on this process. Maintaining elevated cytokinin levels, for instance through the expression of an ipt gene, has been shown to delay leaf senescence and improve drought tolerance by preserving the abundance of proteins involved in energy production and amino acid synthesis (Merewitz et al., 2011). The balance between these opposing hormonal signals—the ratio of ABA/CK, for example—is therefore a critical determinant of the onset and progression of nitrogen remobilization. Other stress-related hormones, such as jasmonates (JA) and salicylic acid (SA), further complicate this network, often cross-talking with ABA and ethylene pathways to modulate defense responses, which can include the reallocation of nitrogen resources away from vegetative growth (Chen et al., 2025; Zhao et al., 2024). Concurrently, the cellular C/N ratio acts as a well-documented global metabolic sensor. An elevated C/N ratio actively triggers a coordinated reprogramming of carbohydrate and amino acid metabolism to restore nutrient balance, a process mediated by ABA and key transcription factors like WRKYs (Diniz et al., 2020).

Secondly, a layer of experimentally supported feedback mechanisms exists where the physiological effects are evident, but the precise molecular sensors remain incomplete. For instance, high levels of sugars in sink tissues can signal strong demand, promoting the expression of transporters and pulling assimilates from the source. Conversely, the accumulation of transport amino acids like glutamine or asparagine in the phloem functions as a negative feedback signal, indicating that sink uptake capacity is saturated and modulating source export accordingly. Furthermore, specific intermediate metabolites, such as 2-hydroxy-5-oxoproline (2HOP), a product of glutamine catabolism, represent an emerging class of systemic signals. 2HOP relies on the plant’s ammonium assimilation status to stimulate nitrate uptake in the roots and coordinate whole-plant nitrogen metabolism (Unkefer et al., 2023). While these metabolic feedback loops clearly tune the supply-demand balance, their specific receptor kinases or binding proteins are largely uncharacterized.

Finally, the most critical yet highly speculative category involves the long-distance signals that directly communicate grain filling status and demand capacity back to the source leaves. While it is conceptually evident that a developing sink exerts a “pull” on resources, the exact molecular messengers transmitting this state are currently hypothesized to include specific phloem-mobile peptides or microRNAs. These putative signals are proposed to travel acropetally to modulate transporter expression in the leaves, but their definitive identification and the characterization of their corresponding perception mechanisms in source tissues remain unachieved.

This finely balanced system is constantly being perturbed by external environmental factors, which provide critical inputs to the regulatory hub. Abiotic stresses such as drought, salinity, and extreme temperatures often trigger a premature senescence program, as the plant prioritizes resource salvage for reproduction over continued vegetative growth. Under drought, for instance, plants not only upregulate ABA synthesis but also initiate a significant metabolic reprogramming, breaking down cellular proteins and channeling the liberated amino acids into compatible solutes like proline for osmotic adjustment, or catabolizing them for energy (Batista-Silva et al., 2019; Kaya et al., 2024). Proteomic analyses of wheat under drought stress reveal a clear upregulation of proteins involved in sucrose and proline biosynthesis, alongside protective LEA proteins, indicating a coordinated effort to manage osmotic stress and protect cellular structures (Silva et al., 2020).

Similarly, other abiotic factors like salt stress trigger a complex response involving the modulation of ion transporters, antioxidant systems, and amino acid metabolism to maintain cellular homeostasis (Li et al., 2021). These stress-induced pathways heavily overlap with developmental senescence, suggesting that plants utilize a conserved toolkit of catabolic and remobilization machinery to respond to a wide range of adverse conditions.

Biotic interactions and exposure to xenobiotics add another layer of complexity, as external agents can directly manipulate the plant’s resource partitioning network. Pathogenic fungi and bacteria often co-opt host nutrient supplies to fuel their own growth. Although mechanistic details are often elucidated in model systems—such as the necrotrophic fungus Botrytis cinerea inducing the amino acid exporter UmamiT20 in *Arabidopsis* to force apoplastic nutrient leakage (Prior et al., 2024)—this hijacking of transport machinery poses a direct threat to the nitrogen pool intended for cereal grain filling. Understanding these pathogen-induced nutrient sinks is crucial for protecting cereal yield. Conversely, beneficial microbes can enhance nutrient mobilization. While frequently studied in legumes like chickpea and soybean (Khan et al., 2019; Jiang et al., 2023), inoculation with plant growth-promoting rhizobacteria (PGPR) offers a promising avenue for cereals as well, potentially enhancing nitrogen metabolism and drought tolerance by modulating plant hormone levels and improving nutrient uptake from the soil. Even agricultural chemicals can have unintended consequences on this network. The herbicide glyphosate, for example, inhibits the shikimate pathway, leading to a drastic perturbation of aromatic amino acid metabolism and cellular redox homeostasis, which in turn affects the abundance of photosynthetic and photorespiratory proteins (Diaz Vivancos et al., 2011). Similarly, exposure to nanoparticles can disrupt central carbon and amino acid metabolism, triggering stress responses that reallocate resources away from growth (Silva et al., 2020; Yuan et al., 2023, 2025).

The intricate synchronization of nutrient flow, governed by this multi-signal integration hub, presents a prime target for genetic improvement of crop nitrogen use efficiency. The natural variation observed in the efficiency of nitrogen remobilization across different genotypes of the same species suggests a rich genetic basis for these regulatory networks (Decouard et al., 2022). Genetic engineering efforts have successfully targeted key components of this system. For example, manipulating the expression of master regulators like NAM-B1 has been shown to directly increase grain protein content in wheat. Similarly, the overexpression of specific amino acid permeases, such as GmAAP6a in soybean, can enhance nitrogen partitioning to the seeds, improving both yield and nutritional quality under low-nitrogen conditions (Liu et al., 2020).

Beyond manipulating transporters and master regulators, engineering specific metabolic pathways has also been explored. For instance, downregulating lysine catabolism or overexpressing key synthesis enzymes has proven effective in creating high-lysine rice, although this can sometimes lead to unintended metabolic trade-offs (Liu et al., 2016; Yang et al., 2018). Because of the complexity and redundancy of the regulatory network, single-gene approaches may have limited success. Future progress will likely depend on a systems-level approach, integrating multi-omics data to identify and manipulate entire regulatory modules or key signaling nodes, rather than individual genes (Turner-Hissong et al., 2020). Understanding how to fine-tune the expression of multiple transporters and metabolic enzymes in a coordinated, tissue-specific manner remains a major challenge and a key frontier in crop science (Milner et al., 2022; Cao et al., 2024).

## 5 Conclusion

In summary, this synthesis underscores that the efficiency of nitrogen remobilization is not a simple sum of source capacity and sink strength, but an emergent property of their tightly coupled relationship. By integrating recent molecular evidence regarding source-leaf metabolic conversion and sink-specific transporter expression, we highlight that optimizing nitrogen use efficiency (NUE) requires understanding a dynamic logistical system rather than manipulating isolated components. This perspective moves beyond a reductionist focus on single genes, elevating the importance of the interfaces between them: the phloem transport network and the signaling cascades that govern it. The senescing leaf is a genetically programmed catabolic engine, and the developing grain is an active, demanding hub; the true nexus of efficiency lies in the precision of their systemic communication.

This synthesis of current literature provides a lens through which apparently conflicting observations can be reconciled. The variable success of single-gene engineering—where overexpressing a transporter yields significant results in one context but creates negative trade-offs in another, such as the unexpected metabolic shifts in high-lysine rice (Liu et al., 2016; Yang et al., 2018)—is the expected outcome of manipulating one component within a complex, non-linear network. The impact of such an intervention depends entirely on whether the targeted gene represents the primary rate-limiting step within that specific cereal genetic background and under those particular environmental conditions.

Despite an advancing conceptual understanding and a growing catalog of molecular players, a critical knowledge gap remains: our ignorance of the long-distance signals that communicate sink demand to the source. We understand that the sink “pulls” nitrogen, but the molecular identity of this signal remains elusive. Without identifying these crucial messengers and their corresponding receptors, efforts to enhance communication between supplying and receiving tissues remain akin to optimizing a supply chain without an inventory management system. To dismantle this barrier, future research must pivot toward a more integrative agenda focused on three key directions:

1. Systematic Identification of Long-Distance Sink-to-Source Signals: A concerted effort must be launched to unmask the mobile signals that communicate sink demand. This requires moving beyond static measurements and employing dynamic experimental systems (e.g., using grafting, de-graining, or targeted sink manipulation) coupled with multi-omics analyses of phloem sap (Niu et al., 2025). Combining deep metabolomics, proteomics, and small RNA sequencing of phloem exudates from contrasting source-sink scenarios will enable the identification of candidate molecules that change in abundance and are transported acropetally. Subsequent functional validation will be essential to confirm their signaling role.
2. Constructing a Spatiotemporal Atlas of the Transport Continuum: To effectively engineer the nutrient transport pathway, we need a high-resolution blueprint of its cellular architecture. The application of single-cell and spatial transcriptomics to the entire source-to-sink axis—from the minor veins of the source leaf, through the stem nodes, and into the maternal and filial tissues of the developing grain—is now feasible. This would generate an unprecedented atlas, revealing precisely which cells express specific transporters, metabolic enzymes, and regulatory factors at each developmental stage, thereby pinpointing the true cellular bottlenecks.
3. Engineering Regulatory Hubs: Armed with a systemic understanding of the signaling network, the focus of genetic improvement should shift from manipulating individual components to fine-tuning the key regulatory hubs that integrate developmental and metabolic signals. Using systems genetics and network analyses to identify these hub transcription factors or signaling proteins (Qu et al., 2019), and employing advanced tools like CRISPR-based promoter editing to modulate their expression in a tissue-specific manner, aims to recalibrate the entire system for higher efficiency. Deciphering the logic of this integrated control system—unmasking its signals, mapping its cellular wiring, and learning to subtly guide its performance—represents a critical frontier in crop science for ensuring sustainable global food security.
